# Detection of hepatitis B virus mRNA from single cell RNA sequencing data without prior knowledge

**DOI:** 10.1371/journal.pone.0314060

**Published:** 2025-02-11

**Authors:** Nicolaas Van Renne, Thomas Vanwolleghem

**Affiliations:** 1 Viral Hepatitis Research Group, Laboratory of Experimental Medicine and Pediatrics, Antwerp University, Antwerp, Belgium; 2 Department of Gastroenterology and Hepatology, Antwerp University Hospital, Antwerp, Belgium; 3 European Reference Network RARE-LIVER, Hamburg, Germany; Centers for Disease Control and Prevention, UNITED STATES OF AMERICA

## Abstract

The ability to detect microbial reads from sequencing data has significantly advanced microbiome and infectious disease research. Recently, INVADEseq introduced a technique to extract microbial reads from single-cell RNA sequencing (scRNA-seq) data following 16S rRNA amplification. We hypothesized that this approach could be leveraged to detect viruses in eukaryotic cells without such amplification or prior knowledge, provided they produce viral mRNAs containing poly-A tails. To test this, we aimed to detect Hepatitis B Virus (HBV) reads from liver samples of patients with chronic HBV infection, both with and without HBsAg loss. We successfully detected HBV reads in the liver of viraemic patients, predominantly in hepatocytes and, to a lesser extent, in Kupffer cells. Functionally cured HBV patients with HBsAg loss had undetectable HBV mRNA in the liver. This study demonstrates the ability to extract and identify viral reads from scRNA-seq data without prior knowledge and without specific amplification. This approach can be used for screening scRNA-seq data for the presence of viral reads at single-cell resolution, potentially enhancing our understanding of the cellular distribution of viruses and virus-host interactions.

## Introduction

Hepatitis B Virus (HBV) continues to be a significant global health challenge, especially in areas with high prevalence of chronic infection. It is a major contributor to hepatocellular carcinoma (HCC), which ranks as the third leading cause of cancer-related deaths worldwide [1]. Studying this virus at the cellular level is crucial for understanding its interaction with its host cell, the hepatocyte, and its impact on immune cells. In fact, detecting viral RNA within individual cells could provide critical new insights into the mechanisms of viral persistence, immune evasion, and disease progression.

Traditional methods for detecting pathogens in sequencing data often require prior knowledge of the specific microbial species present, which limits their utility for discovering unexpected viral infections. However, recently, Galeano-Niño et al. have demonstrated the potential to extract microbial reads from single-cell RNA sequencing (scRNA-seq) data after 16S rRNA amplification [2, 3]. This technique, coined INVADEseq, was initially applied to study the intratumoral microbiota in cancer, revealing significant spatial and cellular heterogeneity influenced by microbial presence. In short, for scRNA-seq, INVADEseq entails the amplification of microbial 16S rRNA sequences that are subsequently co-captured with host RNA within the same droplet. Next, unmapped reads—those reads that do not align to the host genome—are computationally isolated and aligned against a comprehensive reference database of microbial genomes. This process enables the detection and classification of microbial sequences, which can then be assigned back to specific cells, revealing the presence and distribution of both host mRNA and microbial rRNA at single cell resolution.

We hypothesized that a similar approach could be used to detect viral reads in eukaryotic cells, provided the viral mRNAs have poly-A tails. This method does not require a specific amplification step, nor the addition of a ‘viral genome’ to the reference genome for read alignment. Since viral mRNAs are already captured on the beads with their poly-A tails, just like host mRNAs, they should be detectable using the PathSeq [4, 5] algorithm which lies at the core of INVADEseq. This would enable the direct identification and quantification of HBV mRNA from scRNA-seq data, allowing for a detailed analysis of its cellular distribution within liver tissues of patients with chronic HBV infection.

Recently, Narmada et al. published a single-cell landscape of liver from patients with chronic HBV, with and without HBsAg loss [6]. Given that HBV mRNA is polyadenylated, this dataset offers a unique opportunity to test our approach, and explore the cellular distribution of HBV mRNA in the liver of HBV patients.

## Materials and methods

Donor sample generation and processing was described earlier [6]. More specifically, the 10x Genomics Chromium platform with 3’ Gene Expression assay was used to capture single-cell transcriptomes. The raw sequencing data for all available liver samples was downloaded from the NCBI SRA repository (BioProject: PRJNA1096305). This totaled 15 samples from chronic hepatitis B (CHB) patients and 7 from functionally cured (FC) HBV patients.

Raw sequencing reads were mapped on human genome reference (GRCh38-2020-A) using cellranger v7.1.0. This generated the host gene expression raw readcount matrices, and the possorted_genome_bam.bam files containing unmapped reads.

For microbial read detection, a microbe reference files for PathSeq analysis was downloaded from the Broad FTP server (https://software.broadinstitute.org/pathseq/Downloads.html). Next, we downloaded the Visium_pipeline.sh script from the INVADEseq Github (https://github.com/FredHutch/Galeano-Nino-Bullman-Intratumoral-Microbiota_2022/), and adapted it to our single-cell data. We set the PathSeq tool in GATK v4.1.4.1 (https://github.com/broadinstitute/gatk) to process the possorted_genome_bam.bam files with minimum clipped read length (31), and minimum score identity (0.7). In short, this pipeline filters out host reads and aligns the remaining reads to the microbial reference genome, producing output files in CSV formats containing the identified microbial sequences. A custom Python script, INVADEseq.py, was downloaded from the INVADEseq GitHub repository, and executed to link the microbial reads back to specific cells identified in the scRNA-seq data. Thus, generating.csv files that contain raw microbial read counts (S1 Table).

**Fig 1:**
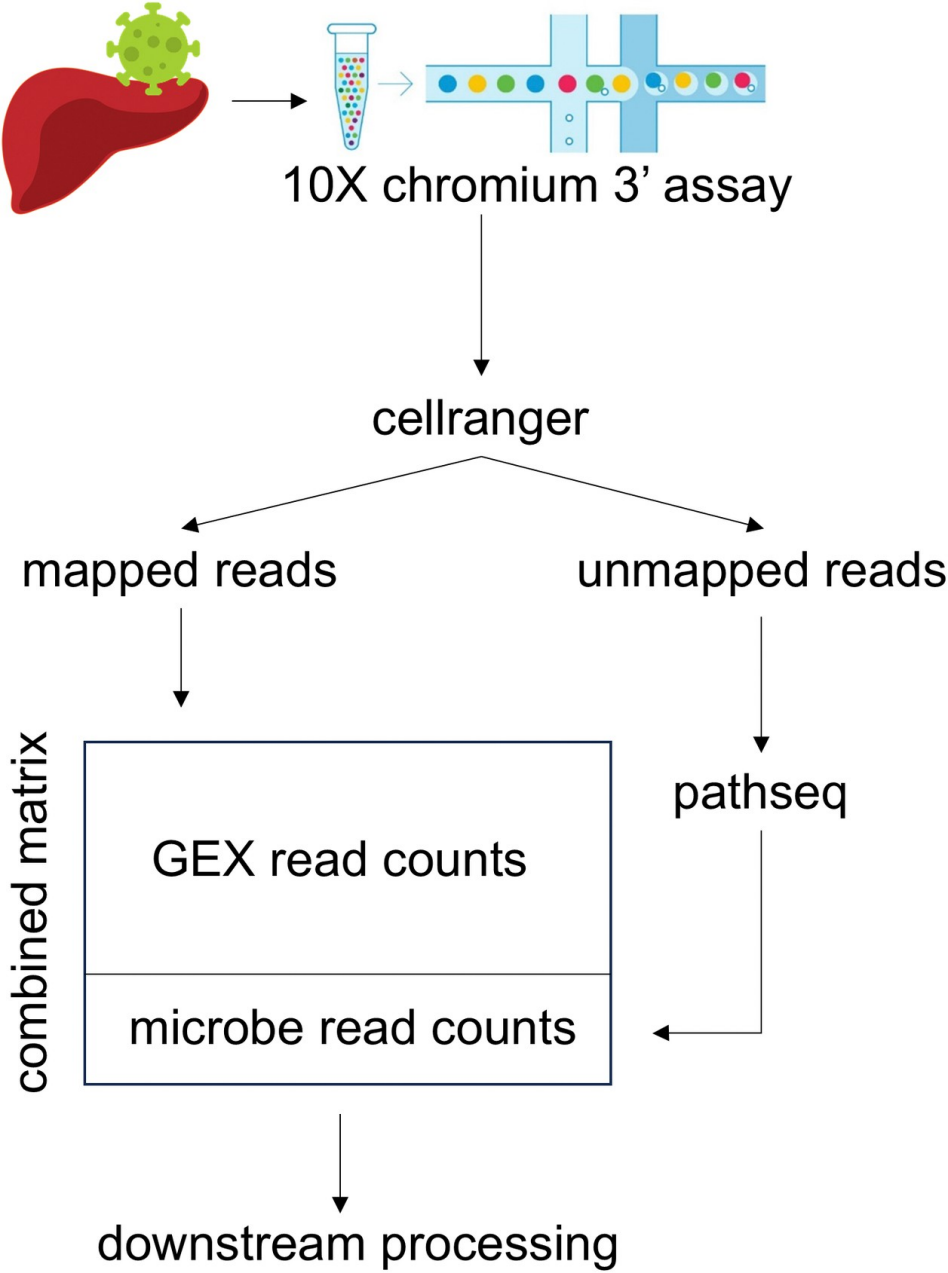
Overview of the pipeline combining host gene expression (GEX) and microbial read detection.

The data matrices containing both host gene expression (GEX) and microbial read data were processed as follows: raw host GEX data were loaded into Seurat (v4.0.2) [7] using the Read10X() function, with cells filtered for number of genes (minimum 200 per cell). Microbial read data were processed to match the structure of the host GEX data matrix, ensuring consistent barcoding and alignment across samples. The microbial and host GEX data were then concatenated into a merged count matrix for each sample, which was re-imported into Seurat. Each concatenated data matrix was then further processed with the Seurat toolbox. In short, to ensure high-quality cell libraries, cells were filtered based on mitochondrial content (20% maximum) and RNA counts per cell (minimum 1,500, maximum 99,999) and minimally 200 different genes per cell. The data were first normalized using the NormalizeData() function to log-transform and scale the counts. Variable features were identified with the FindVariableFeatures() function, selecting the top 2000 most variable genes. The data were then scaled using the ScaleData() function. Principal component analysis (PCA) was performed using the RunPCA() on the first 30 dimensions. Harmony integration [8] was applied to correct for donor effects. The RunUMAP() function was used for dimensionality reduction using the first 30 dimensions. To identify cell clusters, the FindNeighbors() function was called, followed by FindClusters() with a resolution parameter of 1.0. Clusters were annotated using canonical markers. An overview of the pipeline is provided in Fig 1.

### Ethics approval and consent to participate

Not applicable. This study did not involve the collection of new patient data.

## Results

### Cell type clustering and annotation

**Fig 2:**
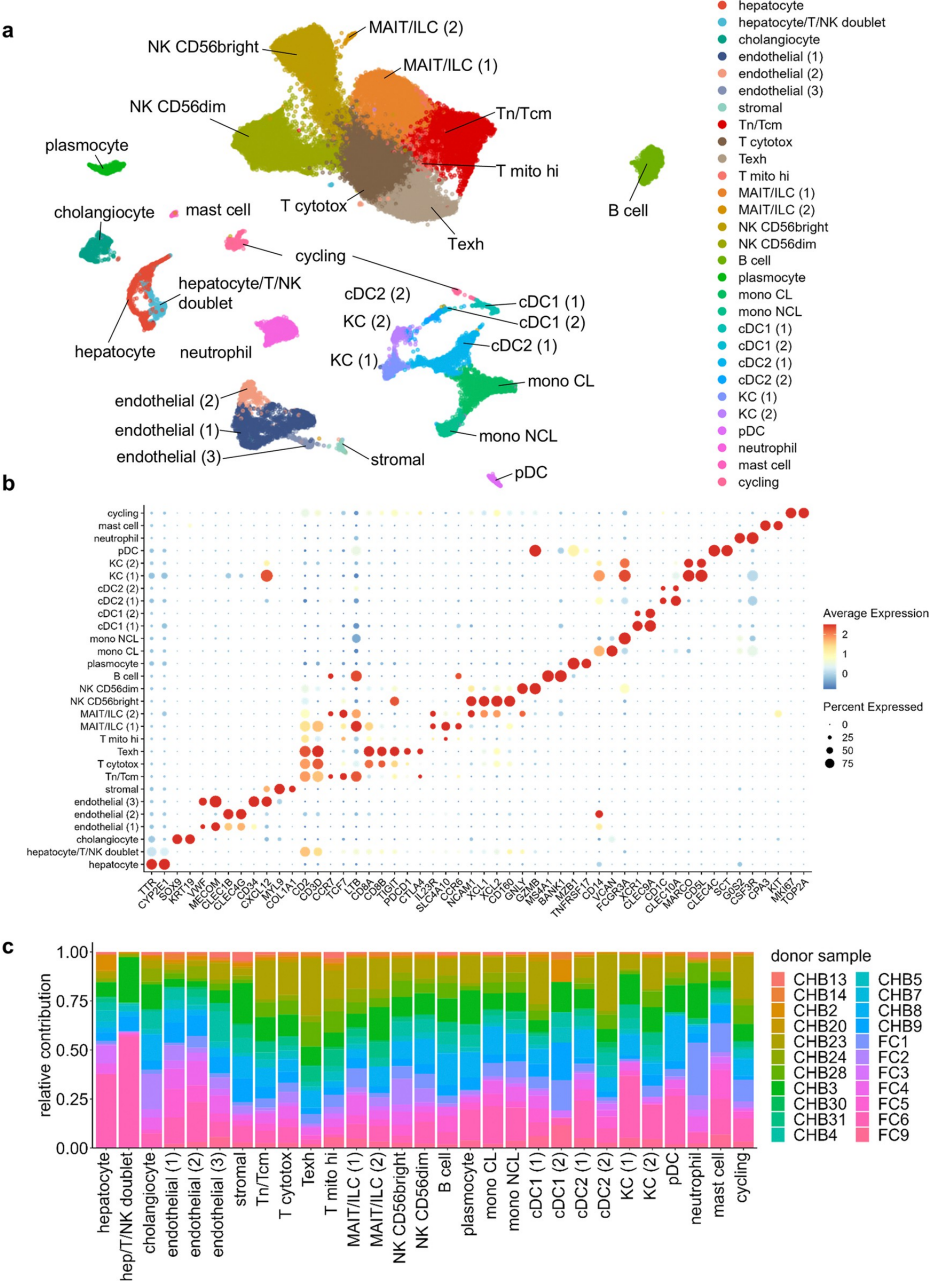
Cell type clustering and annotation. **(A)** UMAP dimensionality reduction plot with annotated cell clusters. **(B)** Dot plots showing marker genes for each cluster. Dot size indicates the proportion of expressing cells, colored by standardized expression levels. **(C)** Relative contribution of each donor sample per cell type cluster. ‘CHB’ indicated chronic hepatitis B, i.e. viraemic patients, while ‘FC’ indicates functionally cured patients, i.e. HBV patients that experienced HBsAg loss. Tn: T naïve, Tcm: T central memory, T exh: T exhausted, MAIT/ILC: Mucosal-associated invariant T or Innate Lymphoid Cells. Mono CL and NCL: classical and non-classical monocyte. cDC and pDC: conventional and plasmacytoid dendritic cell. KC: Kupffer cell.

A total of 39,166 cells were mapped in this study. Samples from chronic hepatitis B (CHB) patients, ie. viraemic patients, accounted for 24,791. The remaining 14,375 cells originated from liver samples of functionally cured (FC) patients, meaning they had experienced HBsAg loss. These cells were grouped into 29 distinct clusters, each of which was annotated based on the expression of canonical marker genes to identify specific cell types (Fig 2A and 2B). To further elucidate the distribution of different patient samples across these clusters, we generated a stacked bar plot (Fig 2C). This visualization highlights the relative contribution of each sample to the identified clusters, and shows that no single sample disproportionately influences the clustering.

**Fig 3:**
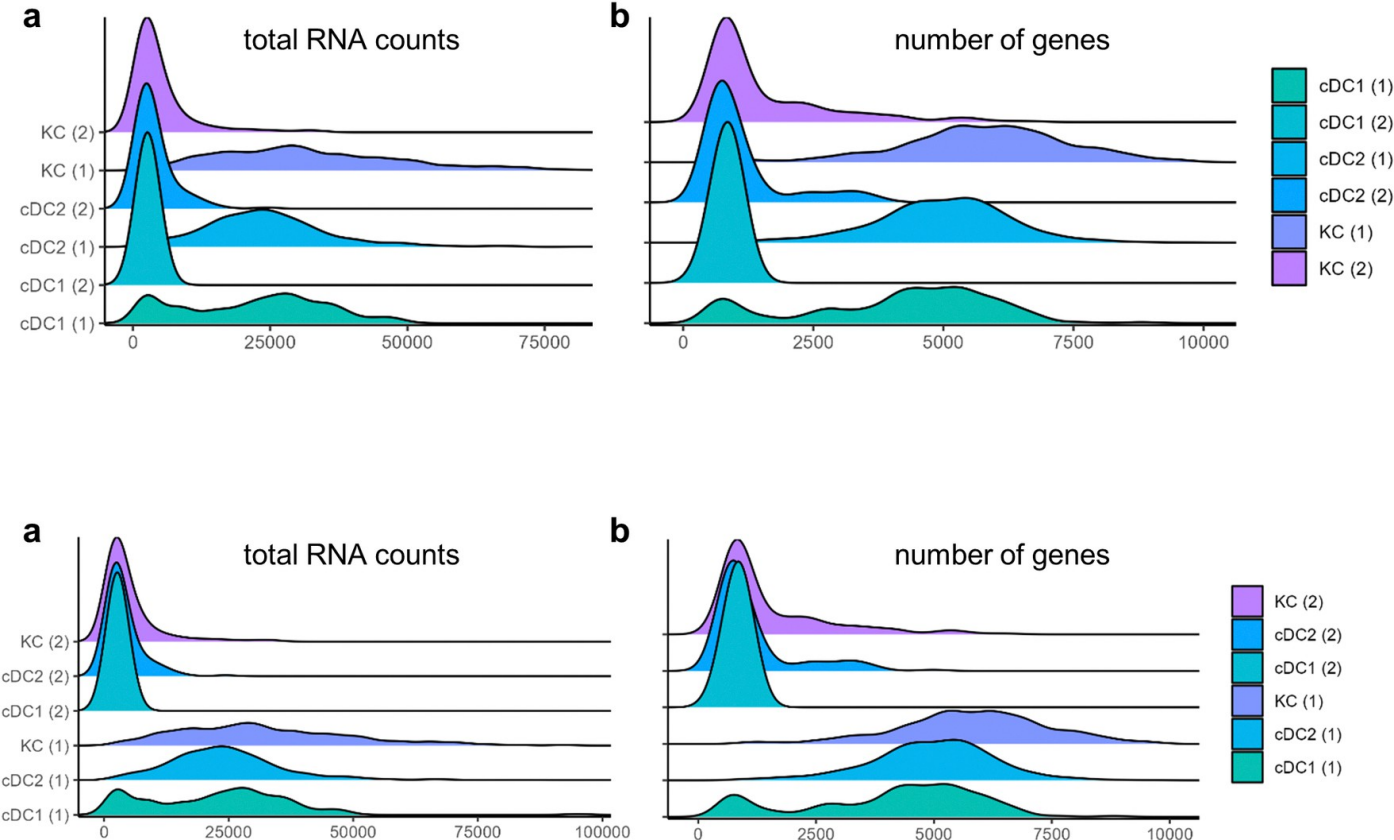
RidgePlots showing total RNA counts and number of detected genes over all cells in the indicated myeloid cell clusters.

Some myeloid celltype clusters, conventional dendritic cell type 1 and 2 (cDC1 and cDC2) as well as Kupffer Cells (KC) were distributed over two clusters. Closer inspection elucidated that the second clusters represented cells with relatively small library sizes. Notwithstanding their low number of detected genes (Fig 3) we were still able to identify these cells through canonical marker gene expression (Fig 2B).

### HBV mRNA detection in individual cells

**Fig 4:**
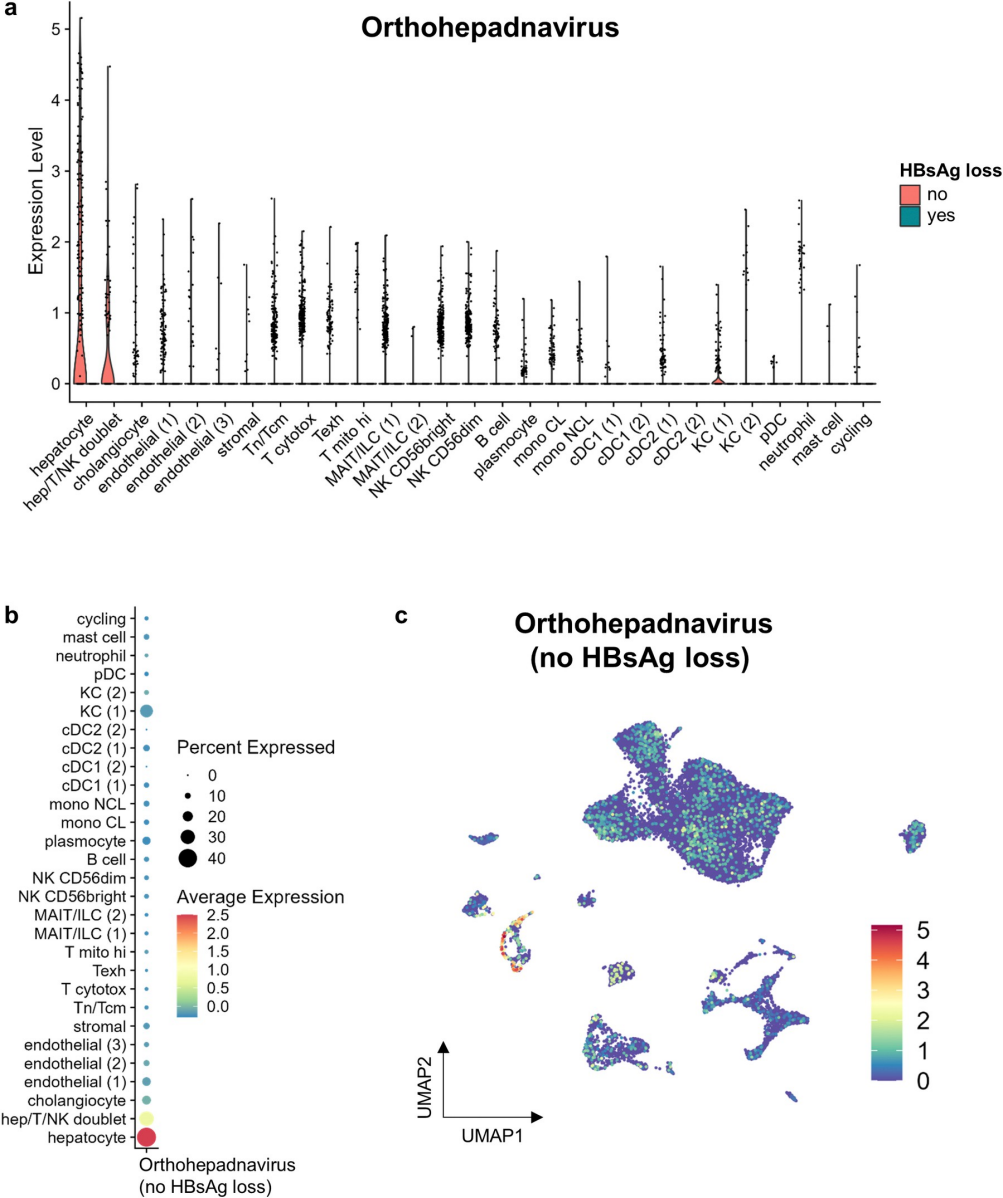
HBV mRNA detection in single cells. **(A)** Violin plots showing expression levels of HBV mRNA split between patients with and without HBsAg loss. Violin plots for patients with HBsAg loss (’yes’) are empty, indicating that HBV mRNA was undetectable in this group. **(B)** Dot plot showing detected levels of HBV mRNA among all cell clusters of patients without HBsAg loss. Dot size indicates the proportion of expressing cells, colored by standardized expression levels. **(C)** UMAP dimensionality reduction plot showing cells with detected HBV mRNA.

After cell clustering and annotation, it was now possible to investigate the distribution of captured HBV mRNA at single cell resolution. We observed that patients who had achieved HBsAg loss exhibited completely undetectable HBV (Fig 4A), indicating the successful clearance of the virus at the cellular level in this patient group. We therefore continued by focusing on the CHB patients with active viraemia. Not unexpectedly, in these liver samples, HBV mRNA was mainly detected in hepatocytes, with up to 42% of all hepatocytes being HBV positive (Fig 4B and 4C). To a smaller extent, hepatocyte/T cell/NK cell doublets were also positive. Notably, other cell types presented low levels of HBV mRNA expression, and this is most likely due to contamination of the cell suspension with reads from hepatocytes. Indeed, hepatocytes are particularly fragile and prone to disintegration during tissue dissociation [9], which results in the release of hepatocyte-specific mRNA into the cell suspension. These mRNAs are then captured by the barcoded beads in droplets containing other cell types. Consequently, non-hepatocyte cells display low levels of hepatocyte-specific gene expression, as shown by TTR and CYP2E1 expression in Fig 2B.

Intriguingly, in these viraemic patients, the KC (1) cluster exhibited a notably higher level of HBV mRNA compared to other non-parenchymal cells, with 26% of these Kupffer cells showing detectable HBV mRNA. This suggests that this may be a level of HBV mRNA that surpasses that of hepatocyte mRNA contamination. As such, Kupffer cells may play an active role in clearing HBV by phagocytosing infected hepatocytes, highlighting a potential mechanism of immune surveillance and viral clearance within the liver.

### Other microbial RNA detection

Finally, marginal levels of bacterial reads were detected (S1 Fig and S1 Table). Importantly, 16S rRNA amplification and capture of the INVADEseq protocol [2] was not performed for this scRNA-seq data set. Hence, these reads may be the result of a specific binding of bacterial transcripts to the capture beads, or they may simply be false positives. In addition, low levels of vesiculovirus RNA were detected. We speculate that these viral reads are false positive signals. In fact, given the stringent requirements for accurate microbial identification, these results should be interpreted with caution, and further validation using complementary methods is necessary to confirm the presence of these microbes.

## Discussion

Our analysis successfully detected HBV reads predominantly in hepatocytes, as expected, given their role as the primary site of HBV replication. However, the detection of HBV reads in other cell types, including immune cells, suggests the possibility of RNA contamination during the dissociation process. Hepatocytes are known to be highly sensitive to lysis, which likely leads to the release of HBV RNA into the extracellular space and its subsequent capture in other cell types during droplet formation. This observation highlights the need for careful interpretation of viral RNA distribution in scRNA-seq data, especially when working with fragile cell types like hepatocytes.

However, the observation that HBV mRNA is detected in over a quarter of the KC (1) cluster of viraemic CHB patients (Fig 4C) is highly suggestive for a true positive detection of HBV mRNA in KCs. KCs may thus participate in HBV clearance by the phagocytosis of infected hepatocytes. Kupffer cells, as liver-resident macrophages, are well-known for their role in immune surveillance and pathogen clearance, but this finding provides direct evidence of their involvement in HBV clearance at the cellular level. This observation could have broader implications for understanding the mechanisms of immune response in chronic HBV infection and might inspire new therapeutic strategies modulating Kupffer cells to enhance viral clearance.

All in all, this study reinforces the feasibility of detecting viral reads from scRNA-seq data, opening new avenues for exploring unknown virus-host interactions at single-cell resolution. The ability to map HBV mRNA to specific cell types within the liver provides an improved level of detail in understanding how viruses interact with their host cells and how the immune system responds to infection. For example, the putative identification of Kupffer cells as active participants in HBV clearance suggests that enhancing the phagocytic activity of Kupffer cells might improve treatment outcomes in chronic HBV patients.

Finally, future studies could extend this approach to other viruses that produce polyadenylated mRNA, potentially leading to new discoveries in virology and immunology.

## Conclusions

This study demonstrates the feasibility and utility of extracting microbial reads from single-cell RNA sequencing data without prior knowledge. This technique was applied to detect HBV mRNA in liver a single cell atlas of HBV patients. HBV mRNA was primarily detected in hepatocytes and, to a minor extent in Kupffer cells. These findings provide new insights into the cellular landscape of chronic HBV infection and highlight the potential role of Kupffer cells in HBV clearance. Further studies are needed to confirm these findings and explore the functional consequences of HBV presence in Kupffer cells.

## Supporting information

S1 Fig Dot plot of microbial read detection. Dot size indicates the proportion of expressing cells, colored by standardized expression levels.
(TIF)

S1 Table Microbial read detection at single-cell resolution of each sample. This table contains the output of pathseq as stored in {each_sample}.gex.filtered_matrix.genus.csv.
(XLSX)

## Abbreviations

HBV: Hepatitis B Virus
CHB: Chronic Hepatitis
B; FC: functionally cured
KC: Kupffer cell
cDC: conventional dendritic cell
UMAP: Uniform Manifold Approximation and Projection
