# Supplementary figures and images for: Detection of hepatitis B virus mRNA from single cell RNA sequencing data without prior knowledge
Source: PLoS One. 2025 Feb 11;20(2):e0314060. doi: 10.1371/journal.pone.0314060 (PMC11813074; doi:10.1371/journal.pone.0314060)

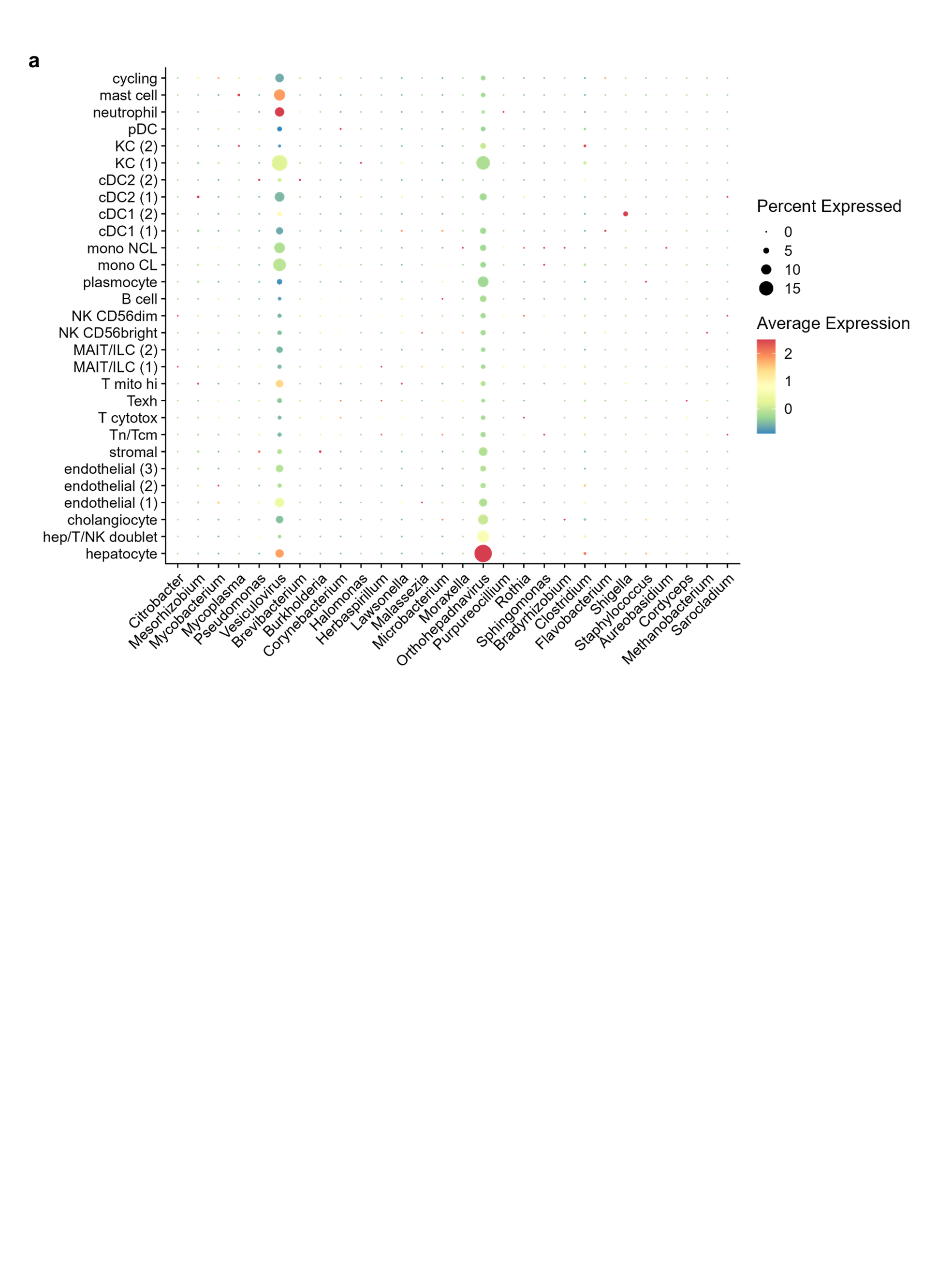

Supplement: S1 Fig — Dot size indicates the proportion of expressing cells, colored by standardized expression levels. (TIF) [file pone.0314060.s001.tif]
